# IgD^+^IgM^−^ B Cells in Common Variable Immunodeficiency

**DOI:** 10.3390/pathogens13020136

**Published:** 2024-02-01

**Authors:** Taissa de M. Kasahara, Sudhir Gupta

**Affiliations:** 1 Department of Microbiology, Immunology and Parasitology, State University of Rio de Janeiro, Rio de Janeiro 21941-853, Brazil; taissakasahara@gmail.com; 2 Division of Basic and Clinical Immunology, Department of Medicine, University of California, Irvine, CA 92697, USA

**Keywords:** B cell, CVID, IgD, tolerance, respiratory allergic diseases

## Abstract

Common variable immunodeficiency (CVID) is the most frequent form of primary hypogammaglobulinemia in adults. In addition to recurrent infections and respiratory manifestations, CVID patients may present several non-infection complications such as autoimmune diseases. The mechanisms that lead to immune dysregulation in CVID are not completely understood. Given the role of IgD on naïve B cells in the maintenance of tolerance and secreted IgD in the respiratory mucosa, we evaluated the frequency of IgD^+^ naïve and IgD^+^ memory B cells in CVID patients. Here, no differences were observed in the percentages and proliferative responses of anergic IgD^+^IgM^−^CD27^−^ B cells between CVID patients, with or without autoimmune disease, and the control group. Interestingly, in the compartment of memory B cells, the percentage of IgD^+^IgM^−^ cells was higher only in CVID patients with allergic rhinitis/allergic asthma. Our results may indicate that anergic IgD^+^IgM^−^CD27^−^ B cells may not be compromised in our CVID cohort. However, IgD^+^IgM^−^ memory B cells may play a role in the immunopathogenesis of allergic rhinitis/allergic asthma in CVID patients. Further studies are needed to better understand the participation of IgD^+^IgM^−^ memory B cells in the immunopathogenesis of allergic rhinitis/allergic asthma in CVID patients.

## 1. Introduction

Common variable immunodeficiency (CVID) is the most frequent form of primary hypogammaglobulinemia in adults and it is characterized by decreased serum immunoglobulin (Ig) G and/or IgA and IgM associated with an impaired response to vaccines [1]. Individuals with CVID present a heterogeneous clinical manifestation and are more susceptible to recurrent infections [1]. Additionally, patients can have non-infection complications such as enteropathy, lymphoproliferation, malignancy, atopy, and autoimmune diseases [2,3,4]. In some cases, these non-infection complications are associated with increased mortality [2,5].

Although monogenic causes have been identified, they represent only a minority of CVID cases [6]. Advances in high-throughput sequencing technologies have improved our understanding of the genetics in CVID pathogenesis [7]. In this context, most cases have a polygenic origin [8]. Furthermore, some mutations have been identified as causative, while others have the potential to predispose or enhance disease severity [7]. The gene penetrance and expressivity, epigenetics, and gene–gene interactions also have an impact on the disease manifestation.

The phenotyping of immune cells, especially B cells, has also contributed to the understanding of CVID. Patients can have a normal or reduced number of total B cells, but most of them have reduced class-switched memory B cells, suggesting an impairment in the activation and differentiation of B cells [9,10,11]. Moreover, an expansion of transitional and CD21^low^ B cells is also observed [9,11]. These alterations have been associated with clinical presentations, including splenomegaly, lymphadenopathy, and autoimmune diseases [9,10,11]. The expansion of CD21low B cells has also been detected in conditions with chronic immune stimulation such as viral infection and autoimmune diseases.

Recurrent respiratory tract infections are the most common clinical feature in CVID, causing sinusitis, otitis media, bronchitis, and pneumonia. In addition to infections, CVID patients present other respiratory diseases such as asthma, bronchiectasis, and interstitial lung disease, which can be consequence of infection or non-infection factors [12,13]. In this context, 15–30% of CVID patients present with allergic rhinitis and allergic asthma [12,14]. Although serum IgE is low in CVID patients, some studies have observed elevated levels of IgE in CVID patients with allergic asthma and other atopic diseases [3,14,15].

During B cell maturation, the B cell antigen receptors IgM and IgD mediate the selection of functional and self-tolerant cells by transmitting survival and activation signals [16]. The elevated expression of surface IgD and downregulation of IgM receptors are hallmarks of anergic naïve B cells that contain autoreactive receptors both in mice and human peripheral blood [17,18]. In these cells, studies have shown that surface IgD restrains the response to self-antigens and promotes the accumulation of anergic B cells [19,20]. Furthermore, the loss of anergic B cells has been observed in some autoimmune diseases [21,22,23].

In addition to the membrane form, IgD can be observed as secreted form in the blood and mucosal surface, especially in the respiratory mucosa [24,25]. Secreted IgD is released by class-switched IgD^+^ B cells; however, its role as a protective or pathogenic component is not clear. Memory B cells that have class-switched to IgD and present an IgD^+^IgM^−^ phenotype are highly reactive to self-antigens in healthy individuals [26]. Additionally, studies have shown increased levels of IgD and tissue IgD^+^ plasma B cells in patients with autoimmune diseases, chronic rhinosinusitis, and atopic asthma [27,28,29,30]. As exposed before, respiratory manifestations and autoimmune diseases are prevalent in CVID patients. However, IgD^+^ naïve and IgD^+^ memory B cells have not been evaluated in these patients. Thus, we investigated the frequency of CD27^−^ and CD27^+^ B cells expressing IgD in the peripheral blood of CVID patients and performed comparisons with their clinical phenotype.

## 2. Materials and Methods

### 2.1. Patients

Peripheral venous blood sample were obtained from 31 patients diagnosed with CVID according to the Pan American Group for Immunodeficiency and European Society for Immunodeficiencies (ESID) criteria [31]. Blood samples were drawn immediately prior to immunoglobulin administration (at trough level). Clinical data were obtained from medical records. As controls, blood samples from 31 age- and sex-matched healthy subjects (18 female and 13 male; mean age of 46.4 years (range 21–76)) were collected. The protocol was approved by the Institution Review Board of the University of California, Irvine and an informed consent was signed by each subject. 

### 2.2. Flow Cytometry Analysis

Peripheral blood mononuclear cells (PBMCs), separated by density gradient centrifugation with Lymphocyte Separation Medium (Life Technology), were resuspended with RPMI medium supplemented with 10% fetal bovine serum, 100 U/mL penicillin, and 100 µg/mL streptomycin at 1 × 10^6^ cells/mL, and then incubated with various combinations of monoclonal antibodies and isotype controls. The following antibodies were used to identify different B cell subsets: anti-CD19-PerCP, anti-CD27-FITC, anti-IgD-BV510, anti-CD38-PE (all from Biolegend, San Diego, CA, USA), and anti-IgM-APC (BD Bioscience, San Diego, CA, USA) (Supplementary Figure S1 and Table S1). 

The cells were acquired using FACSCelesta (Beckton-Dickinson, San Jose, CA, USA) and analyzed using FlowJo software. Isotype control antibodies and single-stained samples were used to periodically check the settings and gates on the flow cytometer. After the acquisition of 100,000 to 200,000 events, lymphocytes were gated based on forward and side scatter properties after the exclusion of dead cells and doublets. 

### 2.3. Cell Sorting and Culture

After PBMC isolation, B cells were enriched by positive selection using magnetic columns according to the manufacturer’s instructions (EasySepTM, StemCell Technology, Vancouver, BC, Canada). The cells were stained with anti-CD19-PerCP-Cy5.5, anti-CD27-PE, anti-IgM-APC, and anti-IgD-FITC (all antibodies from BD Biosciences, San Diego, CA, USA). The B cells, defined as CD19^+^CD27^−^IgD^+^IgM^−^, were sorted with FACSAria II cell sorter (Beckton-Dickinson, San Jose, CA, USA). After sorting, the cells were labeled with 5 µM of CFSE dye (Invitrogen, Eugene, OR, USA) before the culture, according to manufacturer’s instructions. The cells were cultured in AIM-V serum-free medium in the presence of CpG-ODN2006 (InvivoGen, San Diego, CA, USA) (2.5 µg/mL) and anti-CD40 (BD Bioscience, San Diego, CA, USA) (2 µg/mL) in a 96-well U bottom plate at 37 °C in a humidified 5% CO_2_ incubator. On day 5, the cells were harvested and acquired using FACSCelesta (Beckton-Dickinson, San Jose, CA, USA).

### 2.4. Statistical Analysis

Statistical analysis was conducted using the program GraphPad Prism graphic version 6.0 for Windows. The nonparametric Mann–Whitney U test was applied to compare two groups. To compare more than 2 groups, we used the Kruskal–Wallis test followed by Dunn’s multiple comparisons test for data without Gaussian distribution. The significance in all experiments was defined as *p* < 0.05.

## 3. Results

### 3.1. Characteristics of CVID Patients

Characteristics of CVID patients are shown in Table 1. The study group consisted of 31 patients, 20 female and 11 male, with a mean age of 53.9 years (range 13–78). Fifteen patients were diagnosed with autoimmune diseases, including organ-specific autoimmune disease (hypothyroidism and adrenal insufficiency), systemic autoimmune disease (rheumatoid arthritis and anti-neutrophil cytoplasmic antibody (ANCA) vasculitis), and autoimmune cytopenia (autoimmune thrombocytopenia). None of them were on immunosuppressive agents. Thirteen CVID patients without autoimmune disease were diagnosed with respiratory diseases such as allergic rhinitis, allergic asthma, and chronic sinusitis alone or in combination. Two patients had allergic rhinitis and chronic sinusitis, and two patients had allergic rhinitis and asthma. Among the patients with autoimmune diseases, six of them also presented with allergic rhinitis, allergic asthma, or chronic sinusitis. The majority of patients (*n* = 29) were receiving intravenous or subcutaneous immunoglobulin replacement therapy. In regard to B cell percentages, two patients (one with hypothyroidism) had less than 1% of CD19^+^ cells. 

### 3.2. IgD^+^IgM^−^ Naïve B Cells in CVID Patients 

**Figure 1:**
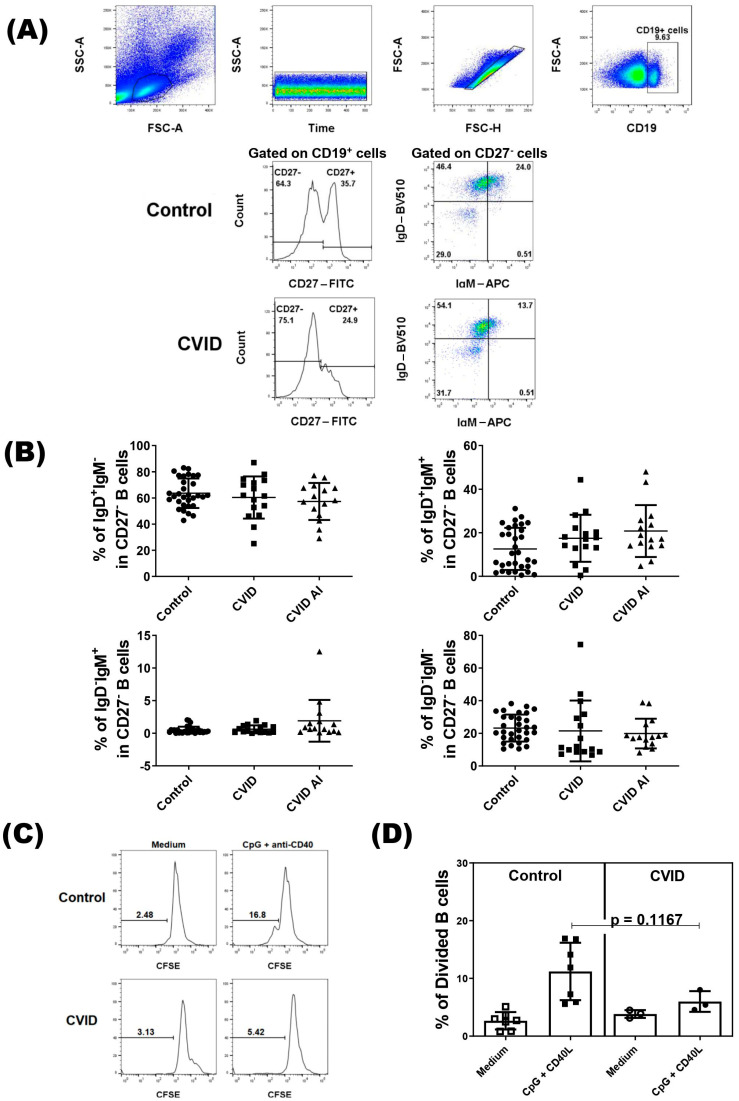
Analysis of CD27^−^ B cells in CVID patients. PBMCs from CVID patients (*n* = 31) and healthy subjects (*n* = 31) were isolated and stained with specific monoclonal antibodies to identify different subsets of B cells according to expression of IgD and IgM. (**A**) Representative dot-plots of CD27^−^ B cell subsets in CVID patients and healthy subjects (control). (**B**) The percentage of CD27^−^ B cell subsets according to clinical presentations of CVID patients. Data are presented as the mean  ±  SD; the *p* values are shown in the figure. Kruskal–Wallis tests followed by the Dunn’s test were applied to compare the groups. (**C**) Representative histograms of CFSE-labeled IgD^+^IgM^−^CD27^−^ B cells in CVID patients and healthy subjects (control). (**D**) The percentage of divided IgD^+^IgM^−^CD27^−^ B cells in CVID patients and controls. Data are presented as the mean  ±  SD; the *p* values are shown in the figure. Mann–Whitney U tests were applied to compare the groups.

Immunophenotyping analysis of B cells was performed following the gating strategy shown in Figure 1A. As IgD^+^IgM^−/low^ naïve B cells contain anergic B cells and reductions in their frequency have been observed in some autoimmune disease, we divided the patients into two groups based on autoimmune manifestations. We did not find difference in percentages of the IgD^+^IgM^−^ subset, or the IgD^+^IgM^+^, IgD^−^IgM^+^, and IgD^−^IgM^−^ subsets in CD27^−^ B cells between control and CVID patients with or without autoimmune diseases (Figure 1B). Regarding the proliferation, we did not observe a difference in the proliferative response of IgD^+^IgM^−/low^ naïve B cells from CVID patients compared with IgD^+^IgM^−/low^ naïve B cells from the healthy subjects (Figure 1C,D).

### 3.3. IgD^+^IgM^−^ Memory B Cells Is Increased in CVID Patients with Respiratory Manifestations

**Figure 2:**
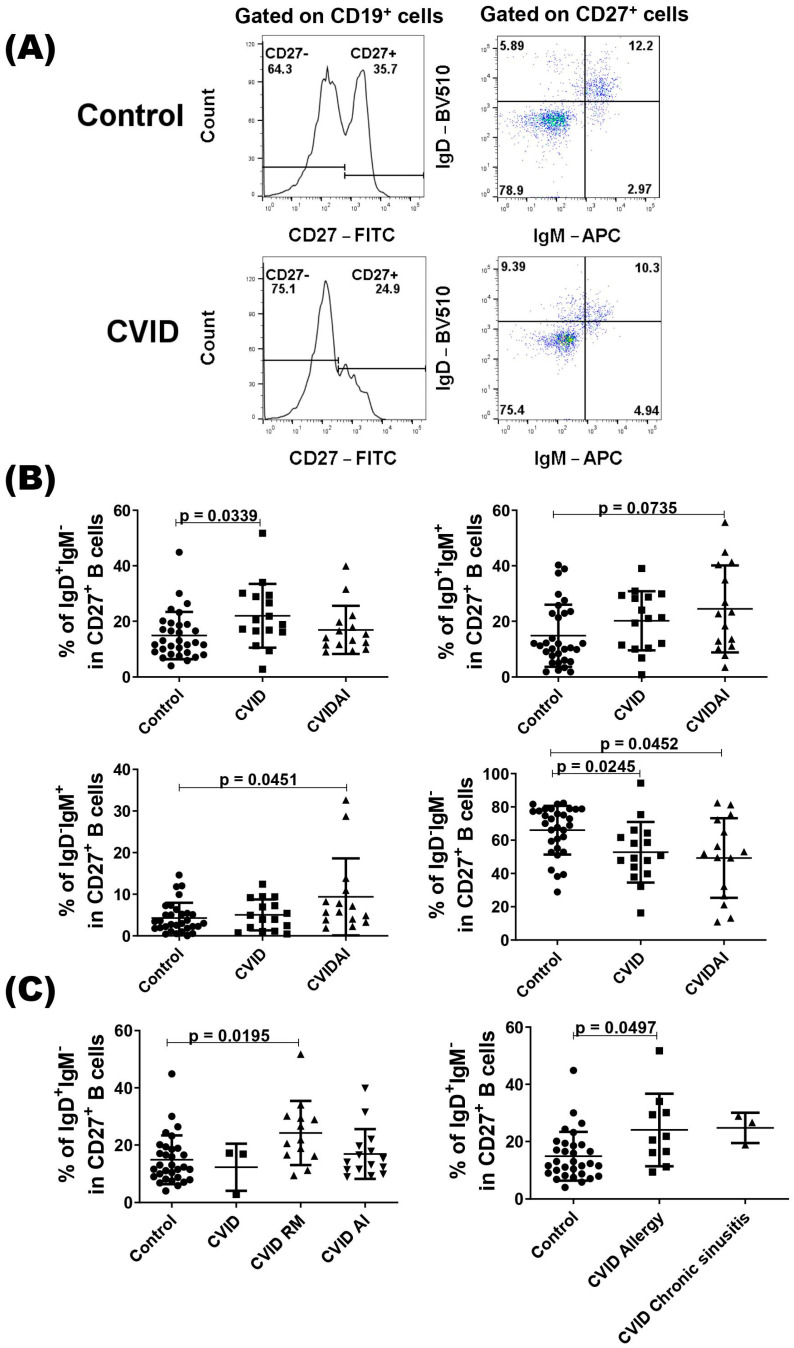
Analysis of CD27^+^ B cells in CVID patients. PBMCs from CVID patients (*n* = 31) and healthy subjects (*n* = 31) were isolated and stained with specific monoclonal antibodies to identify different subsets of B cells according to the expression of IgD and IgM. (**A**) Representatives dot-plots of CD27^+^ B cell subsets in CVID patients and healthy subjects (control). (**B**) The percentages of CD27^+^ B cell subsets according to clinical presentations of CVID patients. (**C**) The percentages of IgD^+^IgM^−^CD27^+^ B cell subsets in CVID patients with autoimmune (AI) or respiratory manifestation (RM) diseases and control. Data are presented as the mean  ±  SD; the *p* values are shown in the figure. Kruskal–Wallis tests followed by the Dunn’s test were applied to compare the groups.

We also evaluated the compartment of CD27^+^ B cells according to the presence or not of autoimmune diseases, and CVID patients with autoimmune disease showed a higher percentage of IgD^+^IgM^+^ and IgD^−^IgM^+^ CD27^+^ B cells compared with the control (Figure 2B). As expected, both patient groups showed a lower percentage of IgD^−^IgM^−^ cells when compared with healthy subjects (Figure 2B). In the IgD^+^IgM^−^ subset, only the group of CVID without autoimmune diseases showed a higher percentage of cell compared with the control group (Figure 2B). As class-switched IgD B cells have been implicated in the respiratory mucosa [32], we evaluated this subset in CVID patients with respiratory manifestations (allergic rhinitis, asthma, and chronic sinusitis) and observed a higher percentage of IgD^+^IgM^−^ CD27^+^ B cells in these patients compared with the control group (Figure 2C). When we divided the patients with allergic rhinitis or allergic asthma and with chronic sinusitis, only patients with allergic manifestations showed a significantly higher percentage of IgD^+^IgM^−^ CD27^+^ B cells (Figure 2C).

## 4. Discussion

CVID is a heterogeneous disease with patients presenting a broad spectrum of clinical manifestations [3]. In addition to recurrent infections, especially of the upper and lower respiratory tract, they can have enteropathy, lymphoproliferation, malignancy, atopy, and autoimmune diseases [3]. Immunophenotyping has helped to understand the immunopathogenesis of this complex disease by identifying defects in B cell development and activation. Furthermore, it enables the patients to be classified into different groups according to their clinical characteristics [9,10,11]. Here, we evaluated the expression of the IgD molecule on naïve and memory B cells from CVID patients and compared it to their associated conditions, which were autoimmune and respiratory manifestations.

Immunoglobulin D (IgD) is mostly known as an antigen receptor of naïve B cells; it is co-expressed with IgM. The regulation of IgD and IgM expression on mature B cells contributes to the tolerance of self-antigens and the prevention of autoimmunity [16]. Autoimmune diseases are a common complication in patients with CVID, including autoimmune cytopenias and organ-specific and systemic autoimmune diseases [4,33]. In our cohort, we observed these three classes of autoimmune diseases. Interestingly, all of them are autoantibody-mediated autoimmune diseases, suggesting that defects in the tolerance mechanisms of B cells may occur in these patients. 

Anergy is one of these tolerance mechanisms, and anergic B cells are characterized as CD19^+^CD27-IgD^+^IgM^−^ in humans, also called BND cells [17,18]. In this context, previous studies have observed a reduction in antigen-specific IgD^+^IgM^−/low^ naïve B cells in patients with autoimmune thyroid disease, type 1 diabetes, and systemic lupus erythematosus [21,22,23]. This reduction was associated with an increased cell activation in early onset and a defect in anergy induction. In our analysis, we did not observe differences in the percentage of IgD^+^IgM^−^ subset in CD27^−^ B cells between patients with or without autoimmune diseases and healthy subjects. However, different from these studies, we did not evaluate antigen-specific B cells; our results may indicate that anergy induction or B cell activation are not compromised in CVID patients. Indeed, the IgD^+^IgM^−/low^ naïve B cells from CVID patients showed the same proliferative response as the cells from the control group.

The analysis of IgD expression together with IgM in our gate strategy allowed for the evaluation of another B cell subset modulated in autoimmune diseases. Increased levels of IgD^−^CD27^−^ B cells, also called double-negative B cells, have been found in autoimmune diseases such as systemic lupus erythematosus and rheumatoid arthritis [34,35]. The increase in this subset was correlated with disease activity, and a reduction was observed during the treatment [34,35]. Here, we did not find differences in the percentages of IgD-IgM^−^ or IgD^−^IgM^+^ subsets in CD27^−^ B cells in patients with or without autoimmune diseases and the control group. The patients in our cohort were not on immunosuppressive agents and the autoimmune disease was not active, which may explain the normal percentage of this cell subset. We did not observe a difference in the percentage of naïve B cells between the groups.

Recently, the role of secreted IgD and the class-switched IgD^+^ B cells in the modulation of immune response has emerged [32]. In humans, IgD^+^IgM^−^ plasma cells are generated in the aerodigestive mucosa, enter the circulation, and migrate to distal mucosal districts, including the middle ear as well as the lachrymal, salivary, and mammary glands [24,25]. In mice, class-switched IgD^+^ B cells have been detected in nasal-associated lymphoid tissue and in submandibular and mesenteric lymph nodes [36]. This suggests an involvement of IgD in mucosal immunity. However, its role as a protective or pathogenic component is not clear, since IgD produced by IgD^+^ B cells binds autoreactive antigens, respiratory pathogens, and environmental antigens [24,26,37]. Studies have shown increased levels of IgD or tissue IgD^+^ B cells in patients with autoimmune diseases, chronic rhinosinusitis, and atopic asthma [27,28,29,30]. In contrast, others have suggested that IgD may play a role in allergen tolerance [37,38].

In our analysis of IgD^+^IgM^−^ subset in CD27^+^ B cells, we observed a higher percentage of this subset in CVID patients without autoimmune diseases, indicating that IgD^+^IgM^−^ memory B cells may not have a role in the presence of autoimmune diseases in CVID patients. Given that IgD has been implicated in the respiratory mucosa [28,29,30], we evaluated this B cell subset in CVID patients with respiratory manifestations. This group was composed of patients with allergic rhinitis, asthma, and chronic sinusitis. We observed a higher percentage of the IgD^+^IgM^−^ subset in CD27^+^ B cells only in CVID patients with respiratory manifestations when compared with the control group. Interestingly, this increase was only significant in patients with allergic manifestations and not in patients with only chronic sinusitis. Studies have observed an increased frequency of IgD^+^ plasma cells and IgD levels in nasal tissues from patients with chronic rhinosinusitis [28,29]. This increase was correlated with eosinophilia and total IgE levels [29], as well as with the presence of pathogenic bacteria [28]. In the context of allergies, an increase in allergen-specific IgD was observed in allergic patients who responded to immunotherapy [37,38]. However, they did not evaluate IgD^+^ memory B cells in these patients or healthy subjects before the treatment [37,38].

The reduction in class-switched memory B cells is frequent in CVID patients, suggesting an impairment in the activation and differentiation of B cells or a low survival of memory B cells caused by increased susceptibility to apoptosis [39,40]. This reduction is also associated with clinical presentation, such as splenomegaly, lymphadenopathy, and autoimmune diseases [10,11,41]. In our patient cohort, we also observed a lower percentage of the IgD^−^IgM^−^ subset in CD27^+^ cells, but no differences between patients with or without autoimmune disease. However, only CVID patients with autoimmune diseases had an increased percentage of IgD^−^IgM^+^ cells. Different from us, some studies have associated a reduction in IgM^+^ memory B cells with recurrent infection and non-infection complications [42,43]. It is important to highlight the heterogeneity of CVID, not only its clinical manifestations, but also its polygenic cause, which can lead to the differences observed here.

One of the limitations of our study is the small number of patients in some groups. Taken together, our results show that anergic IgD^+^IgM^−^CD27^−^ B cells are not altered in CVID patients with or without autoimmune diseases. Therefore, this population of B cells may not be compromised in our CVID cohort. Additionally, future studies may be considered to better understand the participation of IgD^+^IgM^−^ memory B cells in the development of airway diseases in CVID patients and their role in CVID pathogenesis.

## 5. Conclusions

Our results may indicate that anergic IgD^+^IgM^−^CD27^−^ B cells do not appear to play a significant role in autoimmunity associated with CVID, and further studies are needed to verify the participation of IgD^+^IgM^−^ memory B cells in the immunopathogenesis of allergic rhinitis/allergic asthma in CVID patients.

## Figures and Tables

**Table 1:** Characteristics of CVID patients.

Patient	Gender	Age	% of CD19^+^ Cells *	Autoimmune Disease	Respiratory Manifestation
1	F	49	5.61	Autoimmune thrombocytopenia	Allergic rhinitis
2	F	65	2.01	-	Allergic rhinitis
3	F	43	2.72	-	-
4	F	42	11.6	Hypothyroidsm	-
5	M	62	0.43	-	Allergic rhinitis and Chronic sinusitis
6	F	51	0.91	Hypothyroidsm	-
7	M	52	7.28	Hypothyroidsm	-
8	F	23	4.56	-	Chronic sinusitis
9	F	48	8	-	Allergic asthma
10	F	61	11.4	Rheumatoid arthritis	-
11	M	16	8.41	-	Allergic rhinitis and asthma
12	F	57	5.82	ANCA vasculitis	-
13	F	76	4.26	-	Allergic rhinitis and asthma
14	F	61	13.1	Autoimmune thrombocytopenia	-
15	F	61	1.29	-	Allergic rhinitis and Chronic sinusitis
16	M	66	8.29	-	Chronic sinusitis
17	M	13	9.51	-	Allergic rhinitis
18	F	78	4.71	Hypothyroidsm/Rheumatoid arthritis	Allergic rhinitis and asthma
19	F	63	1.73	Hypothyroidsm	Asthma and Chronic sinusites
20	F	70	3.04	-	-
21	F	60	4.29	-	Chronic sinusitis
22	M	72	4.86	-	Allergic asthma
23	M	58	5.01	Autoimmune thrombocytopenia	Chronic sinusitis
24	F	65	2.13	Rheumatoid arthritis	-
25	F	54	2.74	Hashimoto’s thyroiditis	Allergic asthma
26	M	57	8.94	-	Allergic rhinitis
27	M	53	3.93	Adrenal insufficiency	Chronic sinusitis
28	F	45	1.58	Hypothyroidsm	-
29	F	56	9.35	-	-
30	M	73	3.7	-	Allergic rhinitis
31	M	23	6.24	Autoimmune thrombocytopenia	-

* Values obtained from sample blood at the time of recruitment.

## Data Availability

The data presented in this study are available on request from the corresponding author.

## Supplementary Materials

The following supporting information can be downloaded at: https://www.mdpi.com/article/10.3390/pathogens13020136/s1, Figure S1: Dot-plots with the Fluorescence Minus One (FMO) controls; Table S1: List of antibodies used.

## References

[1] Bousfiha A., Jeddane L., Picard C., Al-Herz W., Ailal F., Chatila T., Cunningham-Rundles C., Etzioni A., Franco J.L., Holland S.M. (2020). Human Inborn Errors of Immunity: 2019 Update of the IUIS Phenotypical Classification. J. Clin. Immunol..

[2] Chapel H., Lucas M., Lee M., Bjorkander J., Webster D., Grimbacher B., Fieschi C., Thon V., Abedi M.R., Hammarstrom L. (2008). Common variable immunodeficiency disorders: Division into distinct clinical phenotypes. Blood.

[3] Farmer J.R., Ong M.-S., Barmettler S., Yonker L.M., Fuleihan R., Sullivan K.E., Cunningham-Rundles C., Walter J.E. (2018). The USIDNET Consortium common variable immunodeficiency non-infectious disease endotypes redefined using unbiased network clustering in large electronic datasets. Front. Immunol..

[4] Ho H.-E., Cunningham-Rundles C. (2020). Non-infectious Complications of Common Variable Immunodeficiency: Updated Clinical Spectrum, Sequelae, and Insights to Pathogenesis. Front. Immunol..

[5] Resnick E.S., Moshier E.L., Godbold J.H., Cunningham-Rundles C. (2012). Morbidity and mortality in common variable immune deficiency over 4 decades. Blood.

[6] A Bogaert D.J., Dullaers M., Lambrecht B.N., Vermaelen K.Y., De Baere E., Haerynck F. (2016). Genes associated with common variable immunodeficiency: One diagnosis to rule them all?. J. Med. Genet..

[7] Ameratunga R., Edwards E.S., Lehnert K., Leung E., Woon S.-T., Lea E., Allan C., Chan L., Steele R., Longhurst H. (2023). The Rapidly Expanding Genetic Spectrum of Common Variable Immunodeficiency–Like Disorders. J. Allergy Clin. Immunol. Pract..

[8] van Schouwenburg P.A., Davenport E.E., Kienzler A.-K., Marwah I., Wright B., Lucas M., Malinauskas T., Martin H.C., Lockstone H.E., Cazier J.-B. (2015). Application of Whole Genome and RNA Sequencing to Investigate the Genomic Landscape of Common Variable Immunodeficiency Disorders. Clin. Immunol..

[9] Wehr C., Kivioja T., Schmitt C., Ferry B., Witte T., Eren E., Vlkova M., Hernandez M., Detkova D., Bos P.R. (2008). The EUROclass trial: Defining subgroups in common variable immunodeficiency. Blood.

[10] Warnatz K., Denz A., Drager R., Braun M., Groth C., Wolff-Vorbeck G., Eibel H., Schlesier M., Peter H.H. (2002). Severe deficiency of switched memory B cells (CD27+IgM−IgD−) in subgroups of patients with common variable immunodeficiency: A new approach to classify a heterogeneous disease. Blood.

[11] Piqueras B., Lavenu-Bombled C., Galicier L., Der Cruyssen F.-V., Mouthon L., Chevret S., Debré P., Schmitt C., Oksenhendler E. (2003). Common Variable Immunodeficiency Patient Classification Based on Impaired B Cell Memory Differentiation Correlates with Clinical Aspects. J. Clin. Immunol..

[12] Weinberger T., Fuleihan R., Cunningham-Rundles C., Maglione P.J. (2019). Factors Beyond Lack of Antibody Govern Pulmonary Com-plications in Primary Antibody Deficiency. J. Clin. Immunol..

[13] Patrawala M., Cui Y., Peng L., Fuleihan R.L., Garabedian E.K., Patel K., Guglani L. (2020). Pulmonary Disease Burden in Primary Immune Deficiency Disorders: Data from USIDNET Registry. J. Clin. Immunol..

[14] Agondi R.C., Barros M.T., Rizzo L.V., Kalil J., Giavina-Bianchi P. (2010). Allergic asthma in patients with common variable immunodeficiency. Allergy.

[15] Bjelac J.A., Blanch M.B., Fernandez J. (2018). Allergic disease in patients with common variable immunodeficiency at a tertiary care referral center. Ann. Allergy Asthma Immunol..

[16] Tan C., Noviski M., Huizar J., Zikherman J. (2019). Self-reactivity on a spectrum: A sliding scale of peripheral B cell tolerance. Immunol. Rev..

[17] Duty J.A., Szodoray P., Zheng N.-Y., Koelsch K.A., Zhang Q., Swiatkowski M., Mathias M., Garman L., Helms C., Nakken B. (2009). Functional anergy in a subpopulation of naive B cells from healthy humans that express autoreactive immunoglobulin receptors. J. Exp. Med..

[18] Quách T.D., Manjarrez-Orduño N., Adlowitz D.G., Silver L., Yang H., Wei C., Milner E.C.B., Sanz I. (2011). Anergic Responses Characterize a Large Fraction of Human Autoreactive Naive B Cells Expressing Low Levels of Surface IgM. J. Immunol..

[19] Yasuda S., Sun J., Zhou Y., Wang Y., Lu Q., Yamamura M., Wang J.-Y. (2018). Opposing roles of IgM and IgD in BCR-induced B-cell survival. Genes Cells.

[20] Sabouri Z., Schofield P., Horikawa K., Spierings E., Kipling D., Randall K.L., Langley D., Roome B., Vazquez-Lombardi R., Rouet R. (2014). Redemption of autoantibodies on anergic B cells by variable-region glycosylation and mutation away from self-reactivity. Proc. Natl. Acad. Sci. USA.

[21] Smith M.J., Packard T.A., O’neill S.K., Dunand C.J.H., Huang M., Fitzgerald-Miller L., Stowell D., Hinman R.M., Wilson P.C., Gottlieb P.A. (2015). Loss of anergic B cells in prediabetic and new-onset type 1 diabetic patients. Diabetes.

[22] Smith M.J., Rihanek M., Coleman B.M., Gottlieb P.A., Sarapura V.D., Cambier J.C. (2018). Activation of thyroid antigen-reactive B cells in recent onset autoimmune thyroid disease patients. J. Autoimmun..

[23] Malkiel S., Jeganathan V., Wolfson S., Manjarrez Orduño N., Marasco E., Aranow C., Mackay M., Gregersen P.K., Diamond B. (2016). Checkpoints for Autore-active B Cells in the Peripheral Blood of Lupus Patients Assessed by Flow Cytometry. Arthritis Rheumatol..

[24] Chen K., Xu W., Wilson M., He B., Miller N.W., Bengtén E., Edholm E.-S., A Santini P., Rath P., Chiu A. (2009). Immunoglobulin D enhances immune surveillance by activating antimicrobial, proinflammatory and B cell–stimulating programs in basophils. Nat. Immunol..

[25] Johansen F.-E., Baekkevold E.S., Carlsen H.S., Farstad I.N., Soler D., Brandtzaeg P. (2005). Regional induction of adhesion molecules and chemokine receptors explains disparate homing of human B cells to systemic and mucosal effector sites: Dispersion from tonsils. Blood.

[26] Koelsch K., Zheng N., Zhang Q., Duty A., Helms C., Mathias M.D., Jared M., Smith K., Donald Capra J., Wilson P.C. (2007). Mature B cells class switched to IgD are auto-reactive in healthy individuals. J. Clin. Investig..

[27] Wu Y., Chen W., Chen H., Zhang L., Chang Y., Yan S., Dai X., Ma Y., Huang Q., Wei W. (2016). The Elevated secreted immunoglobulin D enhanced the activation of peripheral blood mononuclear cells in rheumatoid arthritis. PLoS ONE.

[28] Min J.-Y., Nayak J.V., Hulse K.E., Stevens W.W., Raju P.A., Huang J.H., Suh L.A., Van Roey G.A., Norton J.E., Carter R.G. (2017). Evidence for altered levels of IgD in the nasal airway mucosa of patients with chronic rhinosinusitis. J. Allergy Clin. Immunol..

[29] Zhai G.T., Wang H., Li J.X., Cao P.P., Jiang W.X., Song J., Yao Y., Wang Z., Wang Z.Z., Wang M.C. (2018). IgD-activated mast cells induce IgE synthesis in B cells in nasal polyps. J. Allergy Clin. Immunol..

[30] Ohm-Laursen L., Meng H., Chen J., Zhou J.Q., Corrigan C.J., Gould H.J., Kleinstein S.H. (2018). Local clonal diversification and dissemination of B lymphocytes in the human bronchial mucosa. Front. Immunol..

[31] Conley M.E., Notarangelo L.D., Etzioni A. (1999). Diagnostic Criteria for Primary Immunodeficiencies. Clin. Immunol..

[32] Gutzeit C., Chen K., Cerutti A. (2018). The enigmatic function of IgD: Some answers at last. Eur. J. Immunol..

[33] Gereige J.D., Maglione P.J. (2019). Current Understanding and Recent Developments in Common Variable Immunodeficiency Associated Autoimmunity. Front. Immunol..

[34] Jacobi A.M., Reiter K., Mackay M., Aranow C., Hiepe F., Radbruch A., Hansen A., Burmester G., Diamond B., Lipsky P.E. (2008). Activated memory B cell subsets correlate with disease activity in systemic lupus erythematosus: Delineation by expression of CD27, IgD, and CD95. Arthritis Rheum..

[35] Moura R.A., Quaresma C., Vieira A.R., Gonçalves M.J., Polido-Pereira J., Romão V.C., Martins N., Canhão H., Fonseca J.E. (2017). B-cell phenotype and IgD-CD27- memory B cells are affected by TNF-inhibitors and tocilizumab treatment in rheumatoid arthritis van Zelm MC, ed. PLoS ONE.

[36] Choi J.H., Wang K.-W., Zhang D., Zhan X., Wang T., Bu C.-H., Behrendt C.L., Zeng M., Wang Y., Misawa T. (2017). IgD class switching is initiated by microbiota and limited to mucosa-associated lymphoid tissue in mice. Proc. Natl. Acad. Sci. USA.

[37] Shan M., Carrillo J., Yeste A., Gutzeit C., Segura-Garzón D., Walland A.C., Pybus M., Grasset E.K., Yeiser J.R., Matthews D.B. (2018). Secreted IgD Amplifies Humoral T Helper 2 Cell Responses by Binding Basophils via Galectin-9 and CD44. Immunity.

[38] Boonpiyathad T., Pradubpongsa P., Mitthamsiri W., Satitsuksanoa P., Jacquet A., Sangasapaviliya A. (2020). Allergen-specific immu-notherapy boosts allergen-specific IgD production in HDM-sensitized asthmatic patients. Allergy.

[39] Clemente A., Pons J., Lanio N., Matamoros N., Ferrer J.M. (2013). CD27+ B cells from a subgroup of common variable immunodeficiency patients are less sensitive to apoptosis rescue regardless of interleukin-21 signalling. Clin. Exp. Immunol..

[40] Foerster C., Voelxen N., Rakhmanov M., Keller B., Gutenberger S., Goldacker S., Thiel J., Feske S., Peter H.-H., Warnatz K. (2010). B Cell Receptor-Mediated Calcium Signaling Is Impaired in B Lymphocytes of Type Ia Patients with Common Variable Immunodeficiency. J. Immunol..

[41] Sánchez-Ramón S., Radigan L., Yu J.E., Bard S., Cunningham-Rundles C. (2008). Memory B cells in common variable immunodeficiency: Clinical associations and sex differences. Clin. Immunol..

[42] Carsetti R., Rosado M.M., Donnanno S., Guazzi V., Soresina A., Meini A., Plebani A., Aiuti F., Quinti I. (2005). The loss of IgM memory B cells correlates with clinical disease in common variable immunodeficiency. J. Allergy Clin. Immunol..

[43] Blanco E., Pérez-Andrés M., Arriba-Méndez S., Serrano C., Criado I., Del Pino-Molina L., Silva S., Madruga I., Bakardjieva M., Martins C. (2019). Defects in memory B-cell and plasma cell subsets expressing different immunoglobulin-subclasses in patients with CVID and immunoglobulin subclass deficiencies. J. Allergy Clin. Immunol..

