# Supplementary material for: IgD+IgM− B Cells in Common Variable Immunodeficiency
Source: Pathogens. 2024 Feb 1;13(2):136. doi: 10.3390/pathogens13020136 (PMC10891963; doi:10.3390/pathogens13020136)
Supplement: Supplementary file 1 [file pathogens-13-00136-s001.zip › pathogens-2783761-supplementary.pdf]

IgD^{+}IgM^{-} B cells in Common Variable Immunodeficiency

Taissa de M. Kasahara[1], Sudhir Gupta[2*]

[1]Department of Microbiology, Immunology and Parasitology, State University of Rio de Janeiro, Brazil [2]Division of Basic and Clinical Immunology, Department of Medicine, University of California, Irvine, Irvine, CA, United States

*Correspondence: sgupta@uci.edu

Supplementary material

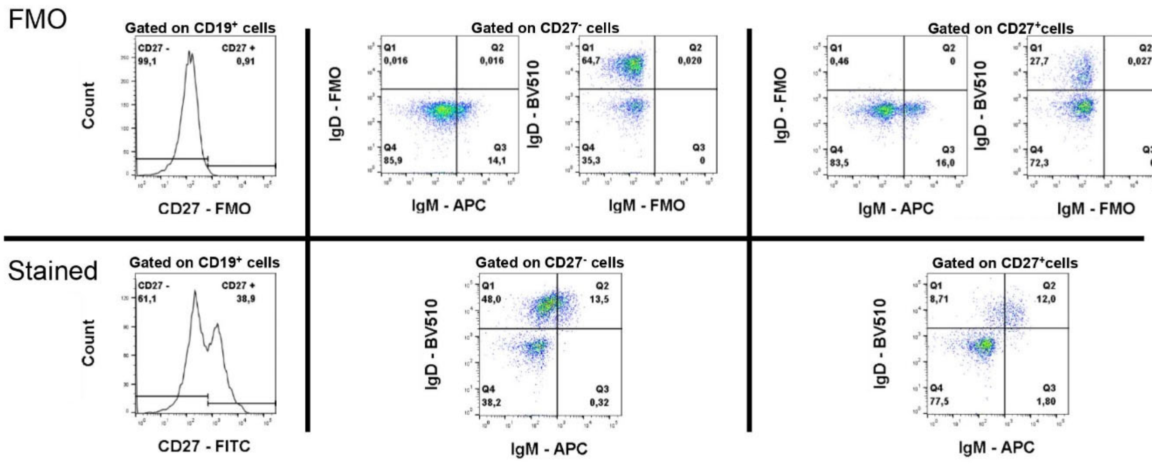

Supplementary Figure S1: Dot-plots with the Fluorescence Minus One (FMO) controls are shown.

Table S1: List of antibodies used.

Marker	Fluorochrome	Company	Cat. number	Clone	Dilution
CD19	PerCP	Biolegend	363014	SJ25C1	1:33
CD27	FITC	Biolegend	302806	O323	1:50
IgD	BV510	Biolegend	563034	IA6-2	1:50
CD38	PE	Biolegend	303506	HIT2	1:50

IgM	APC	BD Bioscience	555441	M-T271	1:15
CD19	PerCP-Cy5.5	BD Bioscience	561295	HIB19	1:50
CD27	PE	BD Bioscience	560985	M-T271	1:50
IgD	FITC	BD Bioscience	562023	IA6-2	1:50
